# Greenlash in the media

**DOI:** 10.1057/s41599-025-05324-7

**Published:** 2025-07-01

**Authors:** Yingnian Tao, Mark Ryan

**Affiliations:** 1 Department of Psychology, Lancaster University, Lancaster, LA1 4YF UK; 2 Department of Marketing, Lancaster University Management School, Lancaster, LA1 4YX UK

**Keywords:** Cultural and media studies, Environmental studies, Language and linguistics, Politics and international relations

## Abstract

This study uses metadata visualisation and corpus linguistics to examine patterns of representation of the emerging term *greenlash* in media reports, as retrieved from the Nexis news database. The metadata analysis reveals that media coverage of greenlash has surged since 2021, predominantly in left leaning European and North American media sources. Through Sketch Engine, collocation analysis identified core thematic areas surrounding greenlash: definition and resistance, status, actor, cause and impact, and location. Our findings indicate that greenlash is primarily a European phenomenon, which may be attributed to a lack of mainstream outlets through which actors can voice opposition to climate policies. The phenomenon is largely driven by economic concerns, in response to specific policies perceived to impose financial burdens on protesting groups. Moreover, our analysis reveals that media organisations often introduce the term greenlash as broad, generalised public opposition to environmental policies rather than a complex, economically driven opposition to specific policies, and portray this opposition in a negative light. We suggest that media organisations may downplay these elements in opposition to neoliberal or populist ideologies or to retain readership. This phenomenon thus highlights the complex intersection between environmental policies, economic burdens, and political divisions underscoring the broader tensions and paradoxes surrounding climate action and socio-economic disparities.

## Introduction

In December 2019, the European Commission launched the European Green Deal, a set of policy initiatives designed to transform the European Union into the first climate-neutral continent by 2050 (Molek-Kozakowska, 2024; Vela Almeida et al. 2023). These initiatives encompass a broad range of policies aimed at addressing climate change, from transitioning to renewable energy to reforming agricultural practices. However, as these policies have begun to be implemented, they have sparked resistance across various countries in Europe. Protests led by farmers, transport workers, and energy-dependent industries have become prominent, with actions in the Netherlands, France, and Germany during 2023 and 2024 (see Chapron, 2024; Cornago, 2023; Matthews, 2024; Tasch, 2024; Tocci, 2023). This pushback reveals the tensions between environmental reforms and socio-economic concerns.

Backlash refers to a reinforcement of existing beliefs when exposed to arguments against these beliefs (Guess and Coppock, 2020). Backlash has historically targeted a range of progressive issues, including immigration (Lesińska, 2014), feminism (Anduiza and Rico, 2024), diversity initiatives (Kidder et al. 2004), political correctness (Read, 2018), and the renewable energy transition. Backlash is often linked to specific policies enabling the success of these movements, where it involves a strong negative reaction with the intention of reversing the targeted policy (Patterson, 2023) or hindering its effectiveness or expansion (Patashnik, 2019). In this study, we only examine the backlash that denotes opposition to, resistance and pushback against environmental policies, agendas and movements, which is broadly labelled as *green backlash*, or *greenlash* for short (Cornago, 2023; Fernandez-Gonzalez et al. 2024).

Despite the increasing use of the term greenlash in global media coverage, especially in Europe, backlash against green policies remains understudied in academic research, with only a handful of studies existing in agriculture (Barbosa de Andrade Aragão et al. 2024), economics (Nguyen, 2022), and climate policy (Fernandez-Gonzalez et al. 2024; Vihma et al. 2021). Also, in the news media, the term greenlash is often used in a relatively vague and generic manner without clearly defining its scope (e.g., what it is against). Therefore, our goal is to shed light on the nuances of how the term is actually used and represented in media coverage in relation to the socio-political dynamics it reflects. This gap is particularly important because media narratives play an important role in shaping public perceptions and influencing policy decisions (Cooper and Nisbet, 2016; Liao, 2023; Shanahan et al. 2011).

This paper addresses this gap by analysing media portrayals of greenlash from across the globe. Using corpus linguistic techniques, it identifies key themes in how greenlash is represented, focusing on the types of resistance, the actors driving the resistance, the causes behind the backlash, the impact, the level of establishment and its location. By examining these global media narratives, this study uncovers the complex intersection of environmental, economic, and political concerns, contributing to a more nuanced understanding of public resistance to environmental policies and its broader implications for governance.

## Backlash against green policies

Green backlash can occur at various levels, from local opposition to clean mobility policies to widespread national protests, such as the Yellow Vest movement in France. These movements often involve specific groups whose livelihoods are directly or indirectly affected by implementation of environmental policies, including farmers and energy sector workers. For example, the 2024 protests across Europe, led by farmers in the Netherlands, France, and Germany, opposed agricultural restrictions and rising energy costs (Chapron, 2024). These protests illustrate how greenlash may manifest as a regional phenomenon, with resistance spreading to neighbouring countries. In some cases, greenlash has prompted political leaders to consider or even roll back environmental policies. Notably, in the summer of 2024, French President Emmanuel Macron and Belgian Prime Minister Alexander De Croo called for a pause in the European green initiatives (Cornago, 2023). Similarly, Italian Prime Minister Giorgia Meloni attributed the severe floods in northern Italy to the challenge of climate policy (Tocci, 2023). These political responses highlight the growing tension between ambitious climate goals and the socio-economic realities faced by certain groups and countries.

Perhaps the most common policies targeted by green backlash are those focused on climate change. Most scientists agree that climate change is happening and that it is at least somewhat anthropogenic in origin (Carlton et al. 2015), yet the climate change issue has been politicised by right-wing political parties to attract voters (Dickson and Hobolt, 2024). In Europe, strong opposition to climate change policies typically only comes from the far right, with mainstream conservative parties generally supporting climate change mitigation policies (Hess and Renner, 2019). In the United States, however, climate change is a partisan issue (Dunlap and McCright, 2008; Zhou, 2016), and a significant portion of Americans deny climate change, including some members of the US government (Boussalis and Coan, 2016; Hilson, 2024). These climate denialists may see climate change as a conspiracy from the liberal elite to benefit globalists by eliminating individual liberties (Vihma et al. 2021). This politicised greenlash view is associated with a strong nationalist ideology (Kulin et al. 2021) and with populism (Huber, 2020), which involves traditional values and desires for strong political leadership. Populism emphasises the contrasts between ordinary people and the elite, and uses climate change as part of “an expression of hostility to liberal, cosmopolitan elites, rather than an engagement with the issue of climate change itself” (Lockwood, 2018, p. 723). In other words, the right-wing populists’ opposition to climate action is more likely attributed to ideological factors than structural economic inequalities (Lockwood, 2018). This leads populist right-wing parties to deny or downplay climate change (Hess and Renner, 2019) and be less likely to emphasise climate protection (Schwörer and Fernández-García, 2024). A similar phenomenon is neoliberalism. In the context of environmentalism, neoliberalism’s stance to climate action is characterised by opposition to regulation that limits innovation in line with the belief that the market will solve the climate problems (Ciplet and Roberts, 2017).

Opposition to green policies may be driven by self-interest. For example, one study found that backlash against electric vehicles occurred in regions that produced parts for gasoline vehicles, but not in regions that received investments for electric vehicles. Likewise, as Cornago (2023) argued, green policies that directly affect the cost of living and carbon taxes are easy to trigger green backlash. Similarly, people who broadly support environmental policies may oppose projects perceived to negatively affect their local communities (Jones and Richard Eiser, 2010) and disrupt their traditional way of life (Fernandez-Gonzalez et al. 2024). Opposition to environmental policies may be linked to economic and employment concerns, fearing job loss or increased costs (Fernandez-Gonzalez et al. 2024; Miniard and Attari, 2021), which have led to protests such as the Yellow Vest movement in France (Tatham and Peters, 2023). People in coal producing regions may demand to be compensated for a shift toward cleaner energy (Gaikwad et al. 2022). Fear, particularly around nuclear energy, is a potential driver of opposition to clean energy policies (Edwards, 2018). People may use protest, lobbying or even violence as a means of opposing green policies, which can lead to policies being weakened, delayed, or cancelled (Sovacool et al. 2022). People also use voting as a tool to punish political groups promoting green policies and projects (Egli et al. 2022; Stokes, 2016). On a positive note, however, resistance to green policies conveys the message that the action towards addressing climate change is no longer politicians’ lip service and lofty ambitions, but is really happening, as people start to pay the price for it (Tocci, 2023).

Although the term greenlash is gaining traction in non-academic sources, it remains underexplored in academic literature. Existing research has primarily focused on opposition to green policies among specific interest groups, particularly farmers, examining their perceptions and motivations for resistance as well as potential mitigation strategies (Barbosa de Andrade Aragão et al. 2024; Howley et al. 2014; Matthews, 2024). Given that greenlash is relatively recent and evolving phenomenon, scholarly attention remains scarce. Within corpus linguistics, opposition to environmental policies has received even less attention. Instead, corpus-assisted discourse studies have predominantly explored broader climate and sustainability discourses in different domains, including corporate sustainability rhetoric, such as fossil fuel companies’ net-zero pledges (Fuoli and Beelitz, 2023); temporal shifts in climate change framing within corporate reports (Ferguson et al. 2016; Jaworska, 2018; Jaworska and Nanda, 2018; Schlichting, 2013); and climate discourse in the media (Bednarek et al. 2022; Gillings and Dayrell, 2024; Huan, 2024). The oil sector has pushed the debate on climate change, making it appear less certain than what the scientific consensus would argue, which may have contributed to a shift in climate change discourse from a scientific matter to an ideological issue (Gupta, 2016). Fashion firms may engage in greenwashing though vague action plans, emphasising a multi-stakeholder approach, and pushing for consumer-facing initiatives (e.g., repair, clean, reuse) to downplay their own responsibilities (Tao and Ryan, 2025). While corpus-assisted research has identified subtle resistance strategies (e.g., appearing vague and uncertain about climate change) in corporate and media discourse, explicit resistance to the climate agenda, such as greenlash, remain understudied. To our best knowledge, no corpus-based study has systematically analysed greenlash, despite its growing media prominence. This gap is particularly salient given the increasing coverage of greenlash, which provides an instant angle to perceive how the media portrays green backlash in the current political environment.

## Data and method

The data used in this study were collected from Nexis, a database for local, national and international general news sources. Previous studies have utilised Nexis in studying societal topics, for instance, the media representation of Muslims (Baker, 2012; Baker et al. 2013), obesity (Baker, 2019; Baker et al. 2020), and climate change (Grundmann and Krishnamurthy, 2010; Nerlich et al. 2012). This database incorporates various types of news, including newspapers, newsletters, web-based publications, weblinks, newswires and press releases, and industry trade press; it also includes magazines, journals and video sources that are transcribed into text, i.e., news transcripts. The comprehensive source types allow us to capture a comprehensive picture of the relatively new phenomenon, which is the primary reason we chose the Nexis database.

We searched the term greenlash in Nexis, setting the Relevance filter to “High”. This was done to ensure the search results focused specifically on articles closely related to the concept of greenlash. We selected all source types under the News category rather than limiting the search to the Newspaper category alone, as greenlash is a relatively new term and media coverage remains limited. By incorporating a broader range of news sources, we aimed to capture a more comprehensive media coverage. As of the end of August 2024, the search of greenlash yielded 120 articles. After excluding 11 non-English articles, we ended up with 109 articles, spanning from 1990 to 2024, with a combined word count of 129,824. All the articles were downloaded as Microsoft Word documents. Then we went through each article to remove non-content materials, such as news outlets’ logos, Nexis metadata (section, length, copyright information), and author information, retaining only the article title, body text, publication date, and news outlets name. After cleaning the data, we converted the data into plain text format and uploaded the files to the online corpus analysis tool Sketch Engine (Kilgarriff et al. 2014), where we compiled them into a Greenlash News Corpus.

The main analyses we conducted were meta-data analysis and corpus queries. Our first step was to analyse the metadata – looking at *when* the term appeared and developed over time, *where* it was published and who was associated with it in news coverage. This helps us map out the broader media landscape surrounding this emerging concept. We obtained the meta-data from Nexis in two ways: by using the platform’s built-in Analytics tab (on the top right corner of search output page) and by downloading the selected files. Next, we will explain how we collected/annotated data for each meta-data type.

**Fig. 1:**
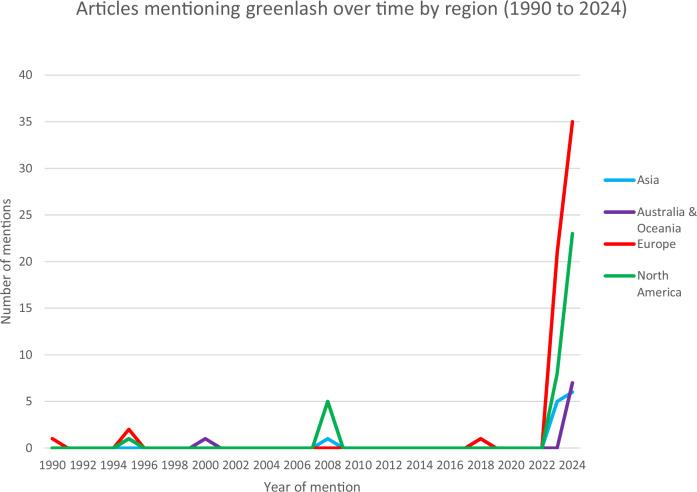
News articles in Greenlash News Corpus over time by region (1990–2024).

To generate information on the date and corresponding mentions of greenlash, we downloaded all the news articles as a single Microsoft Word document. From this Word document, we manually gathered the article titles, dates of publication, and names of news outlets and recorded them in an Excel spreadsheet (Spreadsheet 1). We separately classified news sources into one of four regions (Asia, Europe, North America, or Australia and Oceania) through a manual Google search or by viewing website information. The region of greenlash mentions over time is illustrated in Fig. 1.

We also annotated each news source in Spreadsheet 1 with political stance based on a media bias database called Media Bias/Fact Check (https://mediabiasfactcheck.com/). The majority of news outlets only appear once or twice in our purpose-built corpus. There is only one article from The Telegraph and two articles from CNBC (Consumer News and Business Channel). We present the news outlets with at least 3 articles collected, as seen in Table 1.

**Table 1:** Top publications with more than 2 mentions in the corpus.

News Outlet	Political Stance^*^	Region	Count	Asia	Europe	North America	Australia & Oceania
Financial Times	Centre	Europe	14	0	14	0	0
Newstex Blogs	-	North America, Europe, Asia	14	1	6	7	0
The Guardian	Left-centre	Europe	9	0	9	0	0
National Post	Right-centre	North America	7	0	0	7	0
BBC news	Left-centre	Europe	5	0	5	0	0
The Independent	Left-centre	Europe	5	0	5	0	0
EurActiv.com	Left-centre	Europe	3	0	3	0	0
EuroNews	Left-centre	Europe	3	0	3	0	0
Intellinews	-	Europe, Asia	3	1	2	0	0
Postmedia Breaking News	-	North America	3	0	0	3	0
Yerepouni Daily News EU	-	Europe	3	0	3	0	0
**Count**			69	2.9%	72.5%	24.6%	0%

^*^Ideological stance was taken from a media bias database called Media Bias/Fact Check (https://mediabiasfactcheck.com/). The unlabelled news outlets are not included in this database.

For the prominent people mentioned, we downloaded the meta-data from Nexis containing the name of person and the number of mentions. Then we classified them in terms of their role (e.g., politician, businessperson), representing country/organisation, and the region where they are most active. For instance, Ursula Von der Leyen is most often referred to as the President of the European Commission throughout the corpus, and therefore is annotated as politician for role and EU for location. The result is seen in Table 2.

**Table 2:** Top people mentioned in the corpus.

Top people mentioned	Category	Representing country/Organisation	No. articles mentioned
Ursula Von der Leyen	Politician	European Union	29
Greta Thunberg	Activist	Sweden	10
Rishi Sunak	Politician	United Kingdom	10
Giorgia Meloni	Politician	Italy	10
Donald Trump	Politician	United States	7
António Guterres	Politician	United Nations	7
Justin Trudeau	Politician	Canada	6
Emmanuel Macron	Politician	France	4
Hein Schumacher	Businessman	Unilever	4
Joe Biden	Politician	United States	3
David Johnston	Politician	Canada	2
Narendra Modi	Politician	India	3
Ketanji Brown Jackson	Lawyer	United States	3
Anthony Albanese	Politician	Australia	3
Vladimir Putin	Politician	Russia	2
Boris Johnson	Politician	United Kingdom	2
Olaf Scholz	Politician	Germany	2
Mark Rutte	Politician	Netherlands	2
Sadiq Khan	Politician	United Kingdom	2

This list is based off Nexis’ functionality Top People Mentioned. This is not a comprehensive list of all individuals in the purposely-build corpus. We only included top individuals who are mentioned twice at least.

Each article’s metadata allowed us to track greenlash mentions over time and across regions (Asia, Europe, Australia & Oceania, and North America), revealing regional coverage differences linked to publication sources. The top people influence climate policy and adjust these environmental plans based on the public’s response to these policies.

The second stage of analysis is conducted through corpus queries, including collocation analysis and concordance analysis. Collocation refers to the “process whereby words keep company with one another and thereby convey meaning via co-occurrence” (McEnery, 2006, p.18). In corpus linguistics research, collocation analysis is to study how certain words appear together more often than would be randomly associated. The collocates may appear immediately before or after the word under examination, or at a certain pre-defined distance, e.g., 3 words to the left. The concordance analysis allows users to examine the search word (or term) in more detailed context by displaying the search word in the centre of a line and other words displayed to the left and right (Brookes and Collins, 2024). We obtained collocates of greenlash through the concordance function in Sketch Engine. In addition, to avoid missing other references to green backlash, we included *backlash* and searched for their collocates together with greenlash. Reviewing the context for each collocate enabled us to categorise them thematically, providing a comprehensive view of greenlash. The main thematic categories identified were definition and resistance, status, actor, cause, impact, and location. Details of both analysis stages – meta-analysis and corpus analysis – are expanded upon below.

## Overview of representation of greenlash in news reports: Time, news outlets, and top people

This section provides a metadata-based overview of how the term greenlash is represented in the purpose-built Greenlash News Corpus, focusing on three key factors: temporal distribution, publication sources, and top individuals mentioned. Together, these elements reflect the *aboutness* of the corpus and establish a contextual foundation for the more detailed corpus analysis that follows. By examining when, where and in association with whom greenlash appears, we outline the dynamic change, scope, and political relevance of the discourse.

Figure 1 visualises the number of articles mentioning greenlash over time from 1990 to 2024. Four regions were present in the corpus: Asia, Australia & Oceania, Europe, and North America. Before 2022, greenlash was barely mentioned across the four regions, with only a minor blip in North America (2008) and several scattered mentions in Europe (1995), Australia (1995, 2000), and Asia (2008). However, a sharp increase in mentions began in 2023 for all regions except Australia & Oceania, with Europe leading the trend, reaching 21 mentions in 2023. North America experienced similar growth, although on a smaller scale. Asia and Australia & Oceania also saw increases but at a slower pace. Early data for 2024 suggests that the increase of greenlash mentions will likely to continue, driven largely by publications in Europe and North America. The recent surge could indicate rising global attention as well as scepticism toward green policies, which may result in increased media agenda to cover these topics. Europe’s dominant share of media mentions may point to the continent being at the forefront of implementing ambitious environmental policies, such as the European Green Deal.

**Fig. 2:**
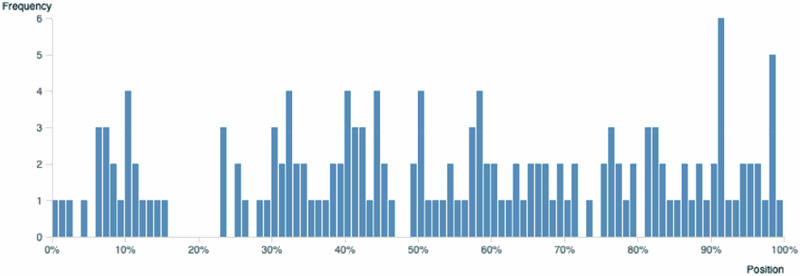
Distribution of *greenlash* in the purpose-built Greenlash News Corpus (granularity set at 100). The chart was created in Sketch Engine.

The mention of the search term greenlash is relatively evenly dispersed in the self-built corpus. As seen in Fig. 2, the blue bar indicates the occurrence of greenlash where it is found. The y-axis indicates the absolute frequency of the occurrence, and the x-axis indicates the parts of the self-built corpus. Except for one section has missing data, the term greenlash generally occurs in all other sections of the corpus, though with some sections more clustered than others.

Table 1 shows the top news outlets that have three or more articles mentioning greenlash, broken down by ideological stance and region. Five out of seven newspapers are identified as left leaning, with only one being identified as right leaning. This highlights a possible ideological skew in greenlash coverage, where centre or left-leaning outlets appear to be more engaged in discussing the topic, which may frame green policies more favourably and view opposition to them as a significant issue. The concordance analysis and collocation analysis in the following section will delve deeper into the framing of greenlash in the news. Regarding region, some news outlets have multiple regions of publications, for instance, the Newstex Blogs are found in three regions: Asia, Europe, and North America. Both Financial Times as newspaper category and Newstex Blogs as blog category are jointly ranked highest, followed by the more general news outlet, The Guardian. The majority of these top news outlets are from Europe (72.5%), followed by North America (24.6%). Interestingly, there is no publication from Australia & Oceania with more than two articles mentioning greenlash. The distribution of regions reflects the dominance of Europe and North America media in coverage of greenlash. Economics-focused news sources, such as the Financial Times, discuss greenlash most frequently among all newspapers and all news types. This might indicate a potential cause of greenlash: the rising cost associated with the green energy transition or green policies. In the following section on themes revealed by greenlash collocates, the economic side of greenlash emerges in the theme Resistance and Cause. Because of its primary focus on financial considerations, it may make more sense to narrow the concept of greenlash to specific types of opposition rather than general resistance to green policies. This point will be discussed further following analyses of greenlash collocates.

In addition to tracking temporal and regional trends and news organisations, we also examined the most frequently mentioned individuals associated with greenlash across the purpose-built corpus. This helps illustrate the global nature of the discourse and highlights the public figures most central to discussions of the phenomenon.

**Fig. 3:**
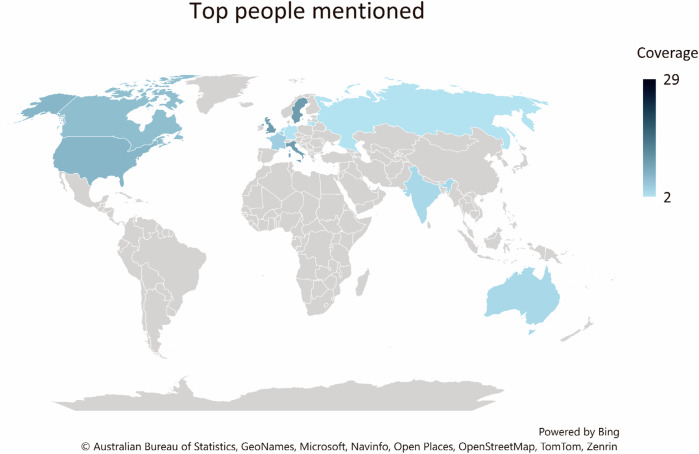
World map of top figures mentioned in Greenlash News Corpus (the darker the colour, the more articles mentioned). Some figures are linked to organisations, rather than countries, and therefore are not represented on the map. The map was created in an Excel spreadsheet based on Table 2.

As seen in Table 2, most of these individuals are high-profile politicians or public figures, including world leaders, activists, and influential businesspersons. Many people listed are politicians from Europe, such as European leaders Ursula Von der Leyen, Rishi Sunak, and Emmanuel Macron. The world map (Fig. 3) highlights that these figures are predominantly based in the EU or North America, with Ursula Von der Leyen as the most frequently mentioned. Most mentions (25/29) of Miss Von der Leyen focus on her efforts to advance the EU Green Deal, showcasing the EU’s green ambitions, for instance, in (1). However, some mentions are critical, noting her adjustments to appease farmer protests. In (2), the article claims that Ms Von der Leyen may scale back the Green Deal approved during her first term just to secure a second term in the European Commission.

1. In the more benign, pre-pandemic and prewar economic environment, mass rallies inspired by green groups and activists such as Greta Thunberg helped to make climate concerns a central electoral issue. In response, mainstream parties were adopting net zero pledges and *Ursula von der Leyen,* the then incoming European Commission president, made the Green Deal – which aims to make the EU climate-neutral by 2050 – her flagship project. (Financial Times, 11 June 2024)
2. Greenlash *Ursula von der Leyen* won her first term by embracing green policies. Her expected bid for a second one involves dumping them, write Andy Bounds and Alice Hancock. (Financial Times, 7 February 2024)

In slight contrast to the mixed sentiments surrounding Ursula von der Leyen, other European leaders are often portrayed hesitant or backtracking on green commitments in answer to public backlash. For instance, in (3), the then UK Prime Minister Rishi Sunak is reported as being vague in banning traditional car sales, while in (4), two European leaders are cited as pausing the EU green agenda.

(3) In the days since Uxbridge, *Rishi Sunak* and his spokesperson can’t seem to come to the same conclusion on whether, for example, the commitment to banning the sale of new petrol and diesel cars by 2030 is still in place. (Independent, 25 June 2023)
(4) The French president, *Emmanuel Macron*, and the Belgian prime minister, *Alexander De Croo*, have both publicly called for a “pause” in the EU’s green legislative agenda, while Poland is fighting for exemptions to sustain its coal subsidies. (The Guardian, 12 July 2023)

Beyond European leaders, figures from North American and Asia such as India’s Narendra Modi are mentioned with mixed sentiments. Joe Biden appears in the context of advocating for more green policies, as seen in (5), whereas Donald Trump is cited for tuning down environmental initiatives.

(5) The *Biden* Administration has showered green energy enterprises, from battery factories to carbon removal projects, with tax incentives. (New York Times, 6 June 2024)

Media coverage of greenlash largely centres on prominent Western policymakers, who either promote or resist environmental policies in response to public reaction. Non-political figures, including activist Greta Thunberg, businessman Hein Schumacher (Unilever), and Supreme Court Judge Ketanji Brown Jackson, are also featured. By featuring both politicians and activists, the news likely reflects the polarisation in public opinion – activists push for stricter environmental policies while politicians, for various reasons, often show resistance. Additionally, certain greenlash collocates (e.g., *homeowners*, *voters*) point to grassroots actors driving opposition to green policies, as explored in the next section.

While this section focuses on prominent individuals – primarily policymakers and public figures who *affect* environmental policy to varying degrees – the following collocation analysis reveals a different group: grassroots actors, such as homeowners and farmers, who are *affected* by and therefore respond actively to environmental policies.

## Thematic categorisation of greenlash collocates

The aforementioned analysis has given an overview of the temporal and regional distribution, publication sources, and top individuals mentioned in the data, which has established the context for further analysis. We believe that the research could be complemented by a more detailed examination of the most prominent collocates of the key terms. Therefore, we move on to elaborate on words that appear together with greenlash and greenlash-related words, i.e., collocates, to reveal themes discussed in the news coverage regarding this phenomenon.

We searched the two lemmas^1^ GREENLASH and BACKLASH that are indicative of opposition to green policies throughout the whole corpus using Concordance functionality in Sketch Engine. Only instances that clearly indicate resistance to general or specific green policies or initiatives were included for further analyses. Eventually, we ended up with 239 instances of greenlash (78 instances for *backlash*, and 161 instances for greenlash). We then collected collocates of all 239 instances with a range of 5 tokens to the left and right. The top 50 strongest collocates are shown in Table 3. Non-words, such as punctuations like comma (,) and hyphen (–), are excluded from the list. These collocates indicate the main aspects of the greenlash phenomenon, such as greenlash as an emerging (*growing*, *emerging*, *coming*) phenomenon that shows resistance (*against*, *resistance*) to environmental policies (*green*, *policies*), greenlash as related to political situations in certain regions (*Europe*, *Europe’s*), and farmers, voters and homeowners as its potential drivers (*voters*, *political*, *homeowner*).

**Table 3:** Top 50 collocates of GREENLASH and BACKLASH.

Collocate	Freq	Salience
against	49	11.86988
growing	18	10.96047
green	36	10.90783
backlash	13	10.40123
or	23	10.34864
policies	18	10.13375
a	96	10.11634
greenlash	11	9.91254
Europe’s	10	9.85432
This	12	9.85364
emerging	7	9.82436
political	12	9.77439
resistance	7	9.74127
termed	6	9.64245
coming	7	9.63743
occurs	6	9.6366
voters	9	9.63077
A	9	9.62303
created	6	9.61918
from	21	9.6126
environmental	9	9.5589
is	42	9.5484
After	6	9.54604
reads	5	9.39119
societal	5	9.38529
In	11	9.33015
Despite	5	9.29956
have	16	9.25013
as	19	9.20764
movement	5	9.1875
which	10	9.16711
The	20	9.15326
results	5	9.12717
Europe	9	9.11464
by	17	9.08044
dubbed	4	9.06926
rise	5	9.05983
homeowners	4	9.05749
over	7	9.05247
age	4	9.0458
has	13	9.02625
prompting	4	9.01701
greener	4	9.01701
driven	4	9.01132
activists	4	8.96658
boilers	4	8.96108
fears	4	8.95561
now	6	8.95561
Guterres	4	8.95015
this	9	8.93391

Salience is the measure of the strength of collocation. In this study, LogDice is used to measure the strength of the collocation. LogDice is not affected by the size of the corpus and can be used to compare scores between different corpora (Gablasova et al. 2017; Rychlý, 2008). The bigger the number, the stronger the association. The maximum value of Log Dice score is 14 and there is no minimum value (Rychlý, 2008). While there is no universal cutoff, a value ≥ 7 is regarded as indicating a strong collocation (Jaworska, 2018).

We further examined these collocates by going through each concordance line, and formulated common themes or patterns associated with greenlash. The results are shown in Table 4. This stage of analysis is more holistic because we examined the full article rather than single lines to get the full context of the topics under discussion. Sometimes, lemmas of different word classes may fall into the same thematic category, such as under the category Resistance, where the words *against* and *resistance* could both be used to convey what greenlash is against. On the other hand, the same collocate may indicate different aspects of the term greenlash and thus fall into different categories. For instance, *political* could be used to refer to the support of greenlash or the cause of greenlash. Collocates used in multiple categories are marked in italics. These categories will be explored in greater detail as follows.

**Table 4:** Top 50 collocates of greenlash.

Category	What it is	Collocates (LogDice)
Definition	What does the term greenlash mean?	*green* (10.91), *backlash* (10.40), or (10.35), *a* (10.12), *greenlash* (9.91), this (9.85), termed (9.64), as (9.21), which (9.17), the (9.15), dubbed (9.07)
Resistance	What is greenlash against?	against (11.87), *green* (10.91), *backlash* (10.40), *policies* (10.13), *greenlash* (9.91), *Europe’s* (9.85), resistance (9.74), environmental (9.56), over (9.05), boiler (8.96), *this* (8.93), *now* (8.96)
Status	What is the status of greenlash?	
Emerging (future)	Emerging phenomenon	growing (10.96), a (10.12), emerging (9.82), *political* (9.77), coming (9.64), *this* (8.93), *now* (8.96)
Existent (present)	Existent phenomenon	have (9.25), age (9.05)
Actor	Who are greenlash supporters?	*Europe’s (9.85), political* (9.77), voters (9.63), from (9.61), *by* (9.08), rise (9.06), homeowners (9.06)
Cause	What causes greenlash?	*Europe’s (9.85), political* (9.77), occurs (9.64), created (9.62), movement (9.19), results (9.13), *by* (9.08), has (9.03), prompting (9.02), driven (9.01), activists (8.97), Guterres (8.95), *now* (8.96)
Impact	How impactful is greenlash?	*Europe’s* (9.85), societal (9.39), despite (9.30), activists (8.97), greener (9.02), fears (8.96), *this* (8.93), *now* (8.96)
Location	Where is greenlash occurring?	*Europe’s* (9.85), in (9.33), Europe (9.11)
Other	Any other category	After (9.55), reads (9.39), is (9.55)

Collocates used in multiple categories are marked in italics.

### Definition and resistance

Collocates under this category indicate what greenlash is about, for example, whether it is resistance to general green policies/agendas or more specific aspects of these policies/agendas. In the news coverage, the types of resistance are often discussed when the term greenlash is first defined; therefore, we incorporate the two categories to elaborate on how greenlash is defined in news coverage. We examined all collocates under Definition and Resistance by manually exploring each concordance line. We found that the majority of definitions of greenlash describe the term as general opposition to environmental/green policies or regulations although there are sporadic mentions of opposition to specific type of green policies or other factors as well.

**Fig. 4:**
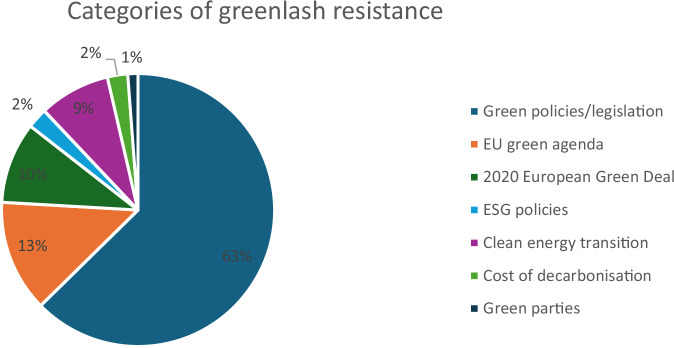
Themes of greenlash resistance.

The most prevalent form of resistance observed in the corpus is opposition to broad environmental policies. As seen in Fig. 4, mentions of green policies/legislation account for over 60% of total references, the EU green agenda accounts for 13%, and the more specific EU green regulations – 2020 European Green Deal – accounts for 10% of the total mentions. Examples (6) and (7) highlight the general pushback, with greenlash defined as backlash against environmental policies or the EU’s broader green agenda. Both instances avoid detailing the specific type of opposition, instead framing greenlash as general resistance against green agendas.

(6) But, since the summer of 2023, the Green Deal has been on regulatory pause, as the EU faces a *“greenlash”* against *environmental policies*. (American Institute for Economic Research, 6 March 2024)
(7) The year was also characterised by a growing *backlash* against the *EU’s green agenda*, driven both by the declarations of some EU leaders and fights within the European Parliament. (Impact Financial News, 13 February 2024)

Apart from the majority sentiment of general opposition, some instances emphasise resistance against a specific environmental policy. For instance, example (8) illustrates resistance to the 2020 EU Green Deal, underscoring that greenlash may manifest more intensively against flagship initiatives with visible socio-economic impacts. The farmers’ protests blocking the Spain-France border serve as an illustration, as they represent farmers voicing their concerns over how the EU Green Deal would affect their livelihoods. This opposition to EU Green Deal aligns with findings in the previous overview section, which show frequent European media reports and mentions of top EU figures.

(8) Recent months have seen a backlash - or *‘greenlash ‘ - against the European Green Deal, t*he main policy of the European Commission under President Ursula von der Leyen: the most visible of these being the farming protests that blocked the border between Spain and France. (EuroNews, 6 June 2024)
(9) The European energy crisis has created a “greenlash,” including *resistance* to *ESG policies,* with parallels in several American red states. (National Post, 10 August 2023)

Another example of resistance to specific green agenda is the UN ESG (environmental, social and governance) policies, as illustrated in (9), showing that this opposition is not exclusive to the EU context. Resistance to ESG policies, while much less frequent than general green policies or the EU Green Deal, appears in North American media, reflecting similar concerns over regulatory and economic implications in the US. Here, opposition to ESG aligns with conservative critiques which view these policies as burdensome or politically driven (for the politicisation of ESG in the US see Hilson, 2024).

Opposition to specific environmental policies also manifests in cost of clean energy. Compared with the general resistance against green policies, references to clean energy transition are relatively more economy oriented, as in (10). The adoption of clean energy, for instance, heat pumps, is significant for homeowners; implementation of a transition to green energy without financial support is likely to receive backlash (Cornago, 2023). In (11), resistance specifically targets wind power, not only due to the high cost of decarbonisation but also through a politically charged narrative. The phrase “wind is woke” frames renewable energy as part of a left-wing agenda, indicating that greenlash is not merely an environmental or economic issue but a politically divisive one. This association of clean energy with political ideology suggests that greenlash can be about value and identity as well as environmental or economic considerations.

(10) Evidence from Europe suggests the “*greenlash” against the high costs of the clean energy transition* has already taken a toll on green parties. (Euro News, 6 June 2024)
(11) To make matters worse, wind developers are facing their own version of the *“greenlash” against the costs of decarbonisation*. The idea that “wind is woke” has taken hold among some critics who view renewables as a partisan leftwing cause. (Financial Times, 22 February 2024)

Resistance as opposition to the green parties is cited once on the corpus. As seen in (12), the 2024 EU election results are described as reflecting the greenlash against green parties as green parties have lost more seats than in the previous election.

(12) On the European front, Miltimore cites the results of the EU parliamentary elections in June together with various regulatory roll-backs both before and after those elections. He (fairly) describes the parliamentary election results as a *‘greenlash***’** against *various Green parties,* and particularly notes the disastrous result for the German Greens: ‘In Germany, the core country of the European green movement, support for the Greens plunged from 20.5 percent in 2019 to 12 percent.’ (Financial Times, 20 June 2023)

In other instances where *green parties* or *greens* are mentioned, they are mostly framed as the impact of greenlash: the green backlash has led Green parties in the European Union and other European countries to lose their seats to competing parties.

### Status

The analysis of collocates reveals that greenlash is represented in relation to the establishment in three ways: as an emerging, ongoing and urgent, or fully established phenomenon. Most frequently, collocates indicate greenlash as an emerging trend gaining traction, predominantly in Europe. Example (13) uses descriptor *growing* as a modifier of greenlash, which signals that it is a nascent force that is starting to affect Europe’s political discourse, specifically right-wing and far-right parties rallying around concerns about the economic impact of environmental policies.

(13) The *growing ‘greenlash’* against Europe’s environmental focus is being fuelled by right-wing and far-right parties across the region, who are attempting to tap into voters’ concerns about the cost of green policies. (Euronews, 6 June 2024)

In (14), *emerging* similarly frames greenlash as an initial yet visible reaction, particularly from populist-right figures who oppose renewable energy. In both examples, the status descriptors (*growing*, *emerging*) are situated as the background, given information, which is used to highlight the foregrounded, new information.

(14) You can see this in the *emerging greenlash* whereby populist-right figures scorn solar and wind farms. (The Conversation, 3 August 2023)

While these examples depict greenlash as gaining momentum, (15) suggests a shift towards urgency. Here, *coming* is part of the predicate, predicting greenlash as an imminent wave rather than as merely a background element. This framing implies an intensified sense of urgency, portraying greenlash as a reactionary force on the horizon that demands attention.

(15) A “*greenlash*” is *coming,* as voters throughout the developed world realise how duped they’ve been by years of unscientific, uneconomic nonsense spouted by much of the media and the so-called “experts”. (Wish Magazine Online Australia, 26 June 2024)

Lastly, some instances describe greenlash as an established movement. Example (16) from The Guardian introduces the *age of greenlash*, indicating that greenlash has evolved from an emerging phenomenon into a well-entrenched movement. In the article’s headline, “Farmers are in revolt and Europe’s climate policies are crumbling. Welcome to the age of ‘greenlash’”, the phrase *the age of greenlash* is used to symbolise the widespread backlash that has emerged, especially in response to European environmental regulations. The first part of the headline, “Farmers are in revolt and Europe’s climate policies are crumbling,” establishes the context and main actors in this backlash. Here, farmers are depicted as rebellious (“in revolt”), and climate policies are described as “crumbling”, implying that farmers’ actions are actively challenging and undermining these policies. Note that farmers (“in revolt”) are juxtaposed with climate policies (“crumbling”). This framing sets up the background for the concluding phrase, “Welcome to the age of ‘greenlash,’“ which amplifies the impact of these protests and weakened policies, suggesting a new era defined by resistance to green initiatives. The title’s critical tone reflects a broader, sceptical view of the greenlash movement, underscoring its perceived influence on current environmental and political dynamics in Europe.

(16) Farmers are in revolt and Europe’s climate policies are crumbling. Welcome to the *age* of ‘*greenlash***’**. Ursula von der Leyen surrendered to angry farmers last week faster than you could shake a pitchfork or dump a tractor-load of manure outside the European parliament. (The Guardian, 16 February 2024)

### Actor

**Fig. 5:**
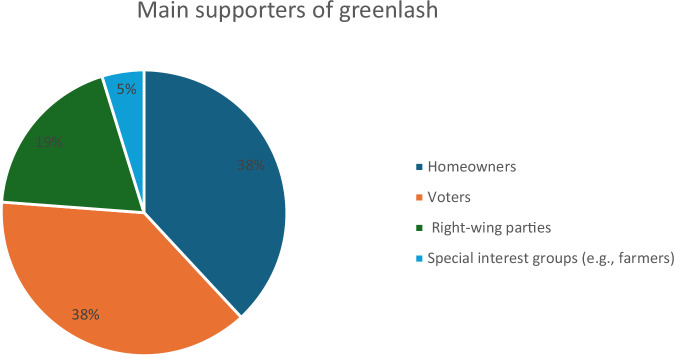
Main supporters of greenlash.

This category explores the primary actors involved in greenlash. We have identified four main groups: homeowners, voters, right-wing political groups, and special interest groups. Figure 5 below illustrates these groups’ support for greenlash.

According to Fig. 5, homeowners, voters and political right-wing groups are the biggest supporters of greenlash, accounting for 95% of all references. Each of these groups has unique but overlapping concerns regarding environmental policies. Homeowners primarily object to the rising cost associated with the clean energy transition. For instance, in Germany, homeowners have protested the high expenses of green boiler installations, which they see as an undue financial burden (17).

(17) Germany last year watered down a proposal on greener boilers after a *backlash from homeowners*, while the EU this year scrapped a target on agricultural emissions after protests from farmers. (Financial Times, 2 June 2024)
(18) The farmer protests and concerns over a *backlash from voters* have already led to a softer tone of the climate roadmap than originally considered, according to people familiar with the matter. (Energy Voice, 6 February 2024)

Voters also play a significant role in greenlash, particularly when their opposition influences political decisions. For example, voter concerns have led to a more flexible approach to EU climate initiatives (18). This demonstrates how public sentiment can moderate the trajectory of environmental policies, as political leaders respond to avoid losing voter support. Right-wing political groups may amplify and seize on homeowners’ and voters’ concerns, channelling them into organised resistance against specific environmental reforms or even against all green policies.

(19) So far, *farmers* have been the most vocal group protesting *against* Europe’s climate policies, and governments have been eager to signal support for them – particularly in rural areas that are bleeding votes to far-right parties. (The Guardian, 31 January 2024)

Special interest groups (Cornago, 2023), for example farmers, have also been vocal in their opposition, as seen in (19). Climate policies may disproportionately impact the livelihood of farmers (Cornago, 2023). Searching the lemma FARMER in the entire corpus has revealed several key grievances: farmers in some countries have been forced to reduce farmland due to nitrogen curbs, scale down livestock production, face competitive disadvantage against non-EU imports, and struggle with rising costs of energy and fertilisers. These four main actors analysed in this section – home-owners, voters, right-wing political groups, and special interest groups – though varied, shared a common concern: environmental policies may impose disproportionate costs on their finances or ways of life.

### Cause

The category Cause explores what motivates greenlash. As shown in Table 5, several drivers of green backlash are identified, including the EU energy crisis, costs associated with the green energy transition, and shifting political dynamics. Among these, energy emerges as a recurring theme, particularly in relation to legislation affecting traditional energy systems. For instance, as seen in (20) and (21), the European energy crisis has spurred resistance to environmental, social, and governance policies, particularly in Germany where rising energy costs have fuelled political opposition. This has led some Germans to support right-wing parties, such as the Alternative for Germany (AfD).

**Table 5:** Categories of cause of greenlash.

Theme	Cause	Mentions
Energy related	A bill to replace gas and oil systems with heat pumps	3
	European energy crisis	2
	Shift to renewable energy	2
	The struggle between fossil fuels and green energy	1
Cost	Cost of going green	1
Multi-factors	Environmental changes	2
	Economic pressures and political dynamics	3
Other	The term Global Boiling	4

(20) The *European energy crisis* has *created a “ greenlash,*” including resistance to ESG policies, with parallels in several American red states. (National Post, 10 August 2023)
(21) Within Germany, *the shift to renewable energy* is causing a political backlash, as consumers are facing higher energy prices and greater uncertainty. This has *created* a “*greenlash*”, encouraging many Germans to turn to right-wing political parties, such as the Alternative for Germany party. (Yerepouni Daily News, 11 September 2023)

Additionally, specific legislation, like Germany’s mandate to replace gas and oil heating systems with heat pumps, has faced backlash (22).

(22) Some governments exacerbated the problem through mis-steps; the botched introduction by Germany’s three-way coalition – including the Greens – of a bill to replace new gas and oil heating systems with heat pumps created a *backlash* exploited by the far-right AfD. (Financial Times, 12 June 2024)

On top of energy and cost-related reasons, other instances refer to political dynamics in the EU and individual European countries, as seen in (23). In (21) and (23), economic pressures and political dynamics interact to drive greenlash across Europe, creating resistance to environmental changes.

(23) This resistance, termed “*greenlash*” is driven by factors ranging from economic pressures to political dynamics. (Intellinews - Romania This Week, 26 January 2024)
(24) Do phrases like this actually help drive us towards faster and more effective climate action? Or do they risk making us prone to climate doomism, and risk prompting a *backlash?* (The Conversation, 3 August 2023)

Interestingly, even the language used to raise climate awareness, like the phrase “global boiling” introduced by UN Secretary-General António Guterres, has faced backlash (24). Critics argue that such a term risks inciting “climate doomism” rather than motivating effective actions. According to a study conducted by Thomson Reuters Institute, overly pessimistic messaging, such as a “doom and gloom” narrative, may cause individuals to disengage (Newman et al. 2019).

### Impact

The *Impact* category assesses the effects of greenlash, particularly within the European political landscape. The reported impact of green backlash varies. A key and immediate effect is the delay or softening of green policies (Patterson, 2023). As seen in (25), mounting resistance has pushed the EU to adopt more lenient climate policies, with Germany diluting its proposal on greener boilers and the EU scrapping certain emissions targets after backlash from farmers (26).

(25) Even before the elections, Ms von der Leyen’s policies on climate action had become more relaxed in response to growing resistance, a phenomenon dubbed the “*greenlash*”. (The Independent, 10 June 2024)
(26) Germany last year watered down a proposal on greener boilers after a *backlash* from homeowners, while the EU this year scrapped a target on agricultural emissions after protests from farmers. Green politicians also suffered big election losses in Europe this month, leading to suggestions that voters are no longer as supportive of ambitious climate policies. (Financial Times, 25 June 2024)

As seen in the previous category *Actor*, *voters* are behind this movement. Backlash against the climate policies could lead to enduring damage to the legitimacy of the policy (McConnell, 2011; Patterson, 2023). In the environment of political election across the Europe, greenlash may lead to a loss of seats for green parties as public support for ambitious climate policies appear to wane.

(27) Although fears of a societal “*greenlash*” – backlash to green policies – are largely unfounded, survey data suggests, climate policies and the Greens have become a focal point for far-right attacks. (The Guardian, 30 April 2024)

In contrast, some sources suggest that fears of widespread societal greenlash are overstated. For example, (27) highlights that, while the far-right may leverage anti-green sentiments, survey data shows enduring public support for climate policies.

(28) Despite the *backlash*, climate change remains at the forefront of European voters’ minds. In last month’s EU Eurobarometer survey on European attitudes on the environment, 78% of respondents said environmental issues had a direct effect on their daily life and 84% agreed EU environmental legislation was necessary for protecting the environment in their country. (BBC, 5 June 2024)
(29) The Public Support Net Zero but the Cost of Living Dominates Despite recent *greenlash*, public concern about climate change and support for action has been growing and that is certainly the case across the EU and the UK. (States News Service, 16 May 2024)

Some instances reinforce that the public support for green policies and initiatives remains unwavering (28) and growing (29) even amidst backlash against green policies. Despite the disruption to green policy implementation, climate change endeavours may remain widely supported.

### Location

The *Location* category addresses where greenlash is most prominent. Through analyses of the collocates *Europe*, *European’s* and *in* (Table 3), it is found that *Europe*, mentioned 23 times, is the focal region, as seen in (30).

(30) In a *continent-wide greenlash, Europeans* are voting against climate zealotry that hikes taxes, inflates the prices of electricity, fuel and food and destroys jobs. (National Post, 11 June 2024)
(31) In *Spain,* one of the EU countries most affected by climate change, the far-right Vox party opposes policies linked to the Green Deal, defining them as “climate fanaticism at the expense of European farmers and ranchers”. (Euronews, 6 June 2024)

Additionally, specific European countries are mentioned too, each with one mention, including Spain, France, Germany, and Poland. Individual countries are often linked to distinct actors or policies fuelling resistance. For example, in Spain, the far-right Vox party criticises the European Green Deal, claiming it exploits farmers and ranchers (31).

## Discussion

Among the top 50 collocates of the key terms GREENLASH and BACKLASH, the ones that refer to the definition and what is opposed to regarding this phenomenon – greenlash – are the most salient collocates, both in terms of their sheer number as well as their saliency parameter. Corpus analyses have revealed that media organisations largely define greenlash as broad public opposition to environmental policies, framing this as a counteraction to climate action and therefore painting a negative, disruptive image of greenlash. The definition of greenlash in our dataset, often but not always, occurs at the very beginning of a news article or the caption of the main images. News organisations tend to introduce greenlash as opposition to overall environmental regulations or policies rather than a specific one (e.g., installing heat pump), serving to denounce or delegitimise any counteraction to climate action. Collocation analysis revealed that the cause of greenlash is primarily economy-driven. However, the economic drivers of this backlash do not appear in the definition but instead are buried deeper within the news reporting, if they are explicitly mentioned at all.

### Economic drivers of greenlash

Our findings reveal that economic concerns are central to greenlash protests. Contrary to media definitions that frame greenlash as blanket opposition to environmental policies, deeper analyses of the actors involved and causes of protests reveal many of these protests stem from specific economic grievances, primarily linked to the economic costs imposed on certain interest groups. For instance, in Germany, homeowners protested against the high costs of transitioning to green boilers, while Dutch farmers resisted nitrogen emission restrictions that constrained their land usage. Moreover, the costs of clean energy transitions, while justified by long-term environmental benefits, impose immediate economic burdens on people. These cases illustrate that greenlash often emerges from groups that perceive these policies as unfair burdens. Arguably, some policies may not be the main contributors of the increased costs, but they become focal points of opposition because of their visibility and immediate economic impacts. For instance, it might be easier for farmers to link economic losses to a policy that tells them they must allocate a certain percentage of their farmland to preserving nature than to broader societal changes, such as technological advances and changes in immigration. This nuanced perspective shows that greenlash is often an immediate economic response to specific policies and therefore may not be an outright rejection of general environmental values.

The fact that economic issues appear to be the drivers of these protests means that some protesters are likely to support environmental policies broadly, only opposing specific policies perceived to have direct economic impacts on the protesting groups. For instance, a recent survey across 125 countries has revealed that over two thirds (69%) of global population are willing to donate 1% of their monthly income to fight for global warming (Andre et al. 2024). An early study found that there are high levels of concern for the environment from both the farmers who are the suppliers of landscape and agricultural goods and the public who are the consumers of countryside amenities (Howley et al. 2014). However, as our findings reveal, people who broadly support environmental policies may oppose projects perceived to negatively affect their local communities (Jones and Richard Eiser, 2010). This is in line with previous research that has found economic factors to be major drivers of farmers’ protests (Matthews, 2024) and of movements such as the Yellow Vest movement in France (Tatham and Peters, 2023), as well as research finding farmers less supportive than the general public of biodiversity initiatives that might reduce the amount of land farmers could use for growing crops (Howley et al. 2014). While it is true that these interest groups are motivated by the financial costs associated with green policies, this does not mean that they oppose the broader goal of addressing climate change.

### Political and regional contexts

Reporting of greenlash has gained popularity mainly in Europe, where the European Green Deal aims to make the European Union climate-neutral by 2050. The impact of this climate agenda is strongest when it actually is implemented, moving from climate goals to climate actions (Tocci, 2023). At the time of media coverage of greenlash, the 2024 European Union election was upcoming, which was a newsworthy topic, especially for European-based news sources. The political dynamics within the bloc and predicted shift to the right following protests against the green agenda are perceived to dominate the election (Abou-Chadi et al. 2024). Unlike in the United States, where mainstream conservative parties openly oppose environmental policies and conservative media channels provide an outlet for opposition (see Hilson, 2024), Europe’s mainstream conservative parties generally support environmental initiatives (Hess and Renner, 2019), which offers fewer mainstream avenues for voicing resistance to green policies. This lack of alternative channels for dissent, along with the increased costs associated with green policies, may have made public protests a primary means of expressing opposition in the EU.

### Media framing and public perception

Overall, although greenlash protests are complex, the centre-left media outlets, which dominate the discussion of greenlash, often define this phenomenon from the lens of opposition to environmental progress with little explicit acknowledgement of the economic grievances underlying much of the backlash. Instead, the nuances of greenlash, such as its economic drivers and opposition to specific policies rather than the environment in general, are downplayed; although many of the media sources surveyed mention them, they generally mention this away from their initial introduction of greenlash. This has the potential to mislead readers, many of whom may not read beyond the definitions of greenlash located early in these articles to explore deeper nuances of the term. Therefore, although liberal newspapers may use more diverse framing when reporting on climate related issues (Dotson et al. 2012), this relegation of these nuanced discussions to deeper sections of articles may cause readers to mistakenly conflate greenlash with broader anti-environmentalism, leading to misunderstandings about the causes of the protests and, as a result, a less favourable perception of the protestors.

This emphasis on environmental policy opposition in definitions of greenlash may be related to the liberal bias that drives many of the media organisations included in this study. Left leaning news organisations may be likely to oppose populist or neoliberal perspectives (e.g., Baker et al. 2020; Brookes and Baker, 2022), including those pushing back against environmental initiatives, and therefore might choose to paint actors opposing environmental policies in a bad light due to their biases or to please their audiences. However, a paradox emerges here, as liberal publications generally also support the economically disadvantaged. As many of the protesting actors fit this description, liberal publications may also want to avoid portraying a negative image of such disadvantaged actors. The choice of these media organisations to frame greenlash from a negative perspective hints that their solution to this paradox involves prioritising environmental issues over social ones. Further investigation could apply lenses such as paradox theory (Smith and Lewis, 2011), which explores competing demands in organisations, for deeper analysis of how media organisations manage contradictory desires.

Furthermore, the nuances of more specific definitions may not be as well received by audiences that hold strong ideological beliefs. The increasing use of A/B headline testing by media outlets (a method of comparing two or more news headline variations to determine the one that engages audiences most, see Hagar and Diakopoulos, 2019) may amplify this bias, as audiences may engage more readily with simple, broadly anti-environmental narratives than with nuanced descriptions of economic and policy-specific opposition. By lumping all kinds of resistance together under a broad umbrella term, protesters may perceive this as ignoring their voices, the very thing that some farmers were protesting against. This simplification risks alienating protest groups who feel their voices and specific concerns are being ignored, which may lead to further backlash. As Sky News reported:

“There are a variety of complaints that you hear, but they boil down to one theme - that they feel left behind, forgotten and disadvantaged. That their role - in feeding the nation - is taken for granted while their problems are ignored.” (Parsons, 2024)

In conclusion, if media organisations want to bring about genuine improvements to the environment, they must understand why these environmental policies have caused so much anger and learn from these reasons to craft better policies that will be more tolerated by society. This may require organisations to put aside their ideological positions, potentially losing readers as a result; however, this is necessary, as a healthy society depends on remaining within the non-negotiable limits of the planet (Desing et al. 2020).

## Conclusion

As a relatively new term, greenlash has gained traction in recent years. This study, through meta-data analysis and corpus analysis, examined how media outlets portray this new phenomenon. Although greenlash was sporadically mentioned as early as 1990, media attention has only surged since 2021, predominantly in Europe and North America media sources with centre-left leanings. The top persons mentioned are primarily European leaders.

While the media organisations tend to depict greenlash as broad public resistance to environmental policies when introducing the new term greenlash, our analysis reveals a deeper economically driven, context-specific cause underlying the phenomenon. This economic underpinning separates greenlash from generalised resistance to environmental agendas, such as anti-environmental movements rooted in climate denial or opposition to environmental protection, which has implications for how governments address this phenomenon. Recognising these nuances is essential for policymakers, who may consider engaging with affected groups to address their economic concerns when implementing environmental policies. By acknowledging the economic and political roots of greenlash, media and policymakers alike can foster a more informed and constructive discourse around environmental initiatives, potentially reducing backlash and increasing public support for sustainable goals.

Our study has limitations. First, the analysis relies exclusively on sources from the Nexis database, which limits our focuses mainly to media outlets rather than broader platforms such as social media. As a result, our findings may not fully capture the spectrum of greenlash discourse across different media types. Second, while Nexis does not intentionally favour one ideological perspective, the mainstream media, especially prominent and internationally recognised outlets, tend to lean centre left. These mainstream media outlets have broad readership and large coverage in Nexis, which may naturally lead to a stronger presence in search results. The representation of greenlash and hence our findings may be biased towards left-wing media. Further studies might want to explore how greenlash is depicted in conservative media sources. Additionally, as greenlash is a relatively new term, media coverage remains sparse and uneven, limiting the breadth of our analysis. As European media sources dominate the coverage while others, such as those in Asian, Australia and Oceania, are underrepresented, which prevents regional comparisons. Our findings are based on existing, limited data, and as political dynamics shift in Europe and beyond, media narratives surrounding greenlash are likely to change. In particular, the re-election of Donald Trump in the United States and the resulting radical shift in direction of the country’s stances on environmental issues has the potential to create ripple effects worldwide, potentially emboldening anti-environmental forces. Future research should account for these changes and explore additional, more balanced news sources to provide a more comprehensive picture of the greenlash phenomenon.

## Data Availability

The data used for this study and corpus query outputs are publicly available on the Open Science Framework via this link: https://osf.io/7ghvc/?view_only=317cc694512540a6acc1fe985b5e5cb2.

## Footnotes

1 Lemma: Lemma is the basic form of a word and is often written in small caps. For example, the lemma DO has several word forms: do, does, did, done, and doing.
